# A Comparison of Computed Tomographic, Radiographic, Gross and Histological, Dental, and Alveolar Findings in 30 Abnormal Cheek Teeth from Equine Cadavers

**DOI:** 10.3389/fvets.2017.00236

**Published:** 2018-01-05

**Authors:** Tiziana Liuti, Sionagh Smith, Padraic M. Dixon

**Affiliations:** ^1^Royal (Dick) School of Veterinary Studies and The Roslin Institute, The University of Edinburgh, Roslin, United Kingdom

**Keywords:** equine dental imaging, equine computed tomography, equine pulpar/apical disease, equine dental pathology

## Abstract

**Background:**

Equine cheek teeth disorders, especially pulpar/apical infections, can have very serious consequences due to the frequent extension of infection to the supporting bones and/or adjacent paranasal sinuses. Limited studies have assessed the accuracy of computed tomographic (CT) imaging in the diagnosis of these disorders, and no study has directly compared imaging and pathological findings of the alveoli of diseased equine cheek teeth.

**Objective:**

To validate the accuracy of CT and radiographic imaging of cheek teeth disorders by comparing CT and radiographic imaging, gross and histological findings in abnormal cheek teeth and their alveoli extracted from equine cadaver heads.

**Study design:**

*Ex vivo* original study.

**Methods:**

Fifty-four cadaver heads from horses with unknown histories that had died or been euthanized on humane grounds obtained from a rendering plant had radiography, CT imaging, and gross pathological examinations performed. Based on imaging and gross examination findings, 30 abnormal cheek teeth (26 maxillary and 4 mandibular) identified in 26 heads were extracted along with their dental alveoli where possible, and further CT imaging, gross, and histological examinations were performed. Eight maxillary cheek teeth (including four with attached alveolar bone) from these heads, that were normal on gross and CT examinations, were used as controls.

**Results:**

Gross pathological and histological examinations indicated that 28/30 teeth, including two supernumerary teeth, had pulpar/apical infection, including pulpar and apical changes. A further supernumerary and a dysplastic tooth were also identified. Abnormal calcified tissue architecture was present in all three supernumerary and in the dysplastic tooth. CT imaging strongly indicated the presence of pulpar/apical infection in 27 of the 28 (96.4%) pulpar/apically infected teeth, including the presence of intrapulpar gas (*N* = 19/28), apical clubbing (*N* = 20), periapical halo (*N* = 4), root lysis or fragmentation (*N* = 7), and periapical gas (*N* = 2). Also present were alveolar bone sclerosis (*N* = 20), alveolar bone thickening (*N* = 3), and lytic/erosive changes (*N* = 8). Radiographic abnormalities strongly indicative of pulpar/apical infection including periapical sclerosis (*N* = 8/28) and apical clubbing (*N* = 14/28) were found in 14/28 (50%) of apically infected teeth. Histological changes were present in alveolar bone of all 21 cases of apical infection where alveolus remained attached to the tooth and was marked in 16 cases, all which had CT alveolar changes. Histological changes included disruption of the normal trabecular pattern, increased osteoclastic activity, and the presence of islands of bone with a scalloped profile within the thickened attached periodontal ligament. No gross pathological or histological changes were present in the eight control teeth or their alveoli (*N* = 4).

**Main limitations:**

No history or breed-related information was available on these cases.

**Conclusion:**

There was a 96.4% correlation between a CT diagnosis and confirmative pathological findings in 28 apically infected teeth confirming the accuracy of CT imaging in diagnosing equine pulpar/apical infections. There was also excellent correlation between CT and histological alveolar bone findings.

## Introduction

Equine cheek teeth disorders, especially bacterial pulpar infection that lead to apical infections (hereafter termed *apical infection*), can be very problematic due to the frequent spread of infection into the mandibles or the maxillary bones and sinuses (1, 2). These infections involve the subgingival aspect of the teeth, which cannot be directly clinically examined, and therefore, imaging including radiography, scintigraphy, and more recently computed tomography (CT) is necessary to assess these disorders. Since treatment is usually by extraction (a major surgical procedure with many potential complications), a definitive diagnosis of apical infection is important before any surgical intervention and imaging contributes significantly to achieving this goal.

Currently, radiography is the main imaging technique used to diagnose equine dental disease, but it is frequently inaccurate in early cases of cheek teeth apical infections (3–9). Scintigraphy also has poor sensitivity (56%) in diagnosing apical infections (4, 5) and is expensive and time consuming. Magnetic resonance imaging requires general anesthesia and is poor at imaging calcified tissues, and thus it has not proven to be reliable in imaging equine dental disorders (10).

Computed tomography (9, 11–14) is more sensitive than radiography in detecting cheek teeth apical infection and is currently the imaging modality of choice for this purpose (9, 14). In addition to detecting lytic or proliferative changes of the alveolar bone and apices of affected teeth (that can often be detected radiographically if advanced enough), CT can detect subtler changes such as root fragmentation, pulp horn irregularities and, especially, the presence of gas [i.e., attenuating structures of −900 to −1,000 Hounsfield unit (HU)] within the pulp or periapical periodontal tissues (9, 13–16).

Several studies (13, 14, 16) have indirectly evaluated the accuracy of CT imaging, primarily by relating clinical and CT findings. A more recent study (9) directly compared CT and radiographic findings with the gross and histological features of 32 extracted apically infected maxillary cheek teeth and showed a 97% correlation between a CT imaging diagnosis of apical infection and the presence of pathological changes of apical infection in the extracted teeth. As significant imaging changes also occur in the alveolar bone of infected teeth, a limitation of the above study of clinical cases is that no alveolar bone was available for pathological examination. The purpose of this study was to examine the alveolar bone of infected teeth for pathological changes where possible to allow comparison with imaging alveolar bone findings, in addition to pathological examination of the extracted teeth to further assess the accuracy of CT and radiographic imaging in the detection of cheek teeth disorders.

## Materials and Methods

This study was approved by the Ethical Review Committee of The Royal (Dick) School of Veterinary Studies and Roslin Institute, The University of Edinburgh on the 16th February 2012. The heads of 54 horses, similar in size to Thoroughbred heads, were obtained from a rendering plant between October 2012 and March 2013. They originated from horses with unknown histories that had died or were euthanized on humane grounds due to disease. The heads were resected at the level of the atlas and then frozen, usually on the day of death.

### Diagnostic Imaging

After they were thawed, six computed radiographic projections were obtained from each head to allow examination of the cheek teeth (Agfa CR 25 digitizer NX8000 HP7900 Raid Server, Agfa Healthcare UK Ltd., Brentford, Middlesex, UK) including: lateral, dorsoventral, two latero 45° dorsal–lateroventral oblique of the maxillary arcades, and two latero 45° ventral–laterodorsal oblique of the mandibular arcades.

Transverse CT images of the heads, positioned on their mandibles, were then acquired with a multislice CT scanner (Siemens, Volume Zoom, Germany) in a helical scan mode using a 512 × 512 matrix, 120 kV, 300 mA, focal spot 1.2, at a slice thickness of 3 and 1.5 mm; images were saved in DICOM format. Radiographic and CT images were evaluated by two observers using dedicated software Osirix^®^ (www.osirix-viewer.com), and a consensus was reached between them. Bone windows (H70) with corresponding window width of 4,000 HU and window level of 1,000 HU were used throughout the evaluation of the CT images. Based on visual examination of incisors and dental imaging examinations, the 26 heads were determined to have a mean age of 16 years (range 5–20 years). Based on imaging findings, followed by a visual dental examination with the jaws disarticulated, 26 heads containing 30 cheek teeth (26 maxillary and 4 mandibular) that were considered abnormal on imaging and/or clinical examination were selected for the study. The 30 abnormal teeth were extracted along with their attached alveoli when possible, using a stainless steel osteotome and mallet. Eight maxillary cheek teeth that were normal on gross examination and on imaging, including four with attached alveoli, were extracted from these heads to serve as controls.

### Comparison of Head Dimensions with Those of Horses of Known Breed

To compare the head sizes in this study population with head sizes of a known breed, CT images of 12 Thoroughbreds of known age [0–5 years old (*N* = 4); 6–15 years old (*N* = 4); and >16 years old (*N* = 4)] were measured to obtain their head dimensions. These Thoroughbreds had undergone CT head imaging for clinical reasons other than sinonasal disorders.

### Head Linear Dimensions and Volumes

Head “length” (i.e., distance from the caudal aspect of the orbit to the nasoincisive notch) was measured from CT sagittal reconstructions; head “width” (width of the hard palate at the level of Triadan 06) was measured from CT dorsal reconstructions, and head “height” (distance from the hard palate to the dorsal aspect of the maxillary bone at the level of the orbit) was measured using CT sagittal reconstructions. These three measurements were multiplied together to produce a measurement of head “volume” (17).

### Evaluation of CT Images

CT evaluation included the assessment of the pulp horns including for the presence of gas-attenuating structures. To accurately identify the presence of gas, a point-shape region of interest (ROI) tool was placed in a consistent manner over the gas-attenuating structures within the pulp horn, and the HU value was generated as shown in Figure 9. The ROI size was identical for all abnormal pulps. Density values between −900 and −1,000 HU were considered to represent gas. CT assessment also included examination of the calcified apical tissues, identification of gas in the apical periodontal membranes, and evaluation of the periapical alveolar bone changes including for the presence of lysis and thickening.

### Gross Anatomical Examination

Each extracted abnormal and control tooth was photographed on all sides and grossly examined, before being transversely sectioned into four equal parts, i.e., occlusal, two mid-crown and apical aspects, using a 1 mm thick lapidary blade. A 5 mm thick section was cut from each of the above portions, then visually examined and photographed on both sides. These thin sections were fixed in formalin before decalcification, histological processing, and examination.

### Histological Processing and Examination

The dental sections were decalcified and embedded in paraffin wax, and 4 µm thick sections were cut and stained with hematoxylin and eosin as described (9). All abnormal and control teeth and their histological slides were anonymized, relabeled, and examined by two observers. Histological examination of each of the four sections of each tooth documented findings in: pulp; primary, secondary, and tertiary dentine (if present); infundibulae (in maxillary cheek teeth); peripheral cementum; periodontal ligament; and alveolar bone. Recorded pulpar changes included the presence of intrapulpar bacteria, infiltrates of neutrophils, lymphocytes and/or plasma cells, necrotic or “faded pulp” (9), and pulp stones. All enamel was lost during decalcification.

### Statistical Analyses of Head Sizes

A paired *T*-test was used to assess for differences in dimensions between the cadaver heads used in this study and the 12 control Thoroughbred horses. No significant difference was found between length (*p* = 0.196), width (*p* = 0.942), height (*p* = 0.086), or volumes (*p* = 0.829) of the two groups of heads.

## Results

Since gross pathological and histological changes are the gold standard for confirming the presence of dental disease, the pathological findings are presented first here, in contrast to the sequence of the actual study where imaging was initially performed.

Thirty abnormal cheek teeth were identified including 26 maxillary teeth: Triadan 06 (*N* = 6); 07 (*N* = 1); 08 (*N* = 4); 09 (*N* = 7); 10 (*N* = 3); 11 (*N* = 2); and 12 (supernumerary teeth) (*N* = 3), and 4 mandibular teeth: Triadan 08 (*N* = 1); 09 (*N* = 2); and 11 (*N* = 1).

### Gross Pathological Findings in Extracted Teeth

#### Alveolar Bone

Alveolar bone surrounding the dental reserve crown and apex remained intact around 21 teeth (all with apical infection) but became detached from the other 9 teeth during extraction or histological processing. Gross examination of the external aspects of the dental alveoli was unrewarding due to iatrogenic chisel damage from the extraction process.

#### Pulp

**Figure 1:**
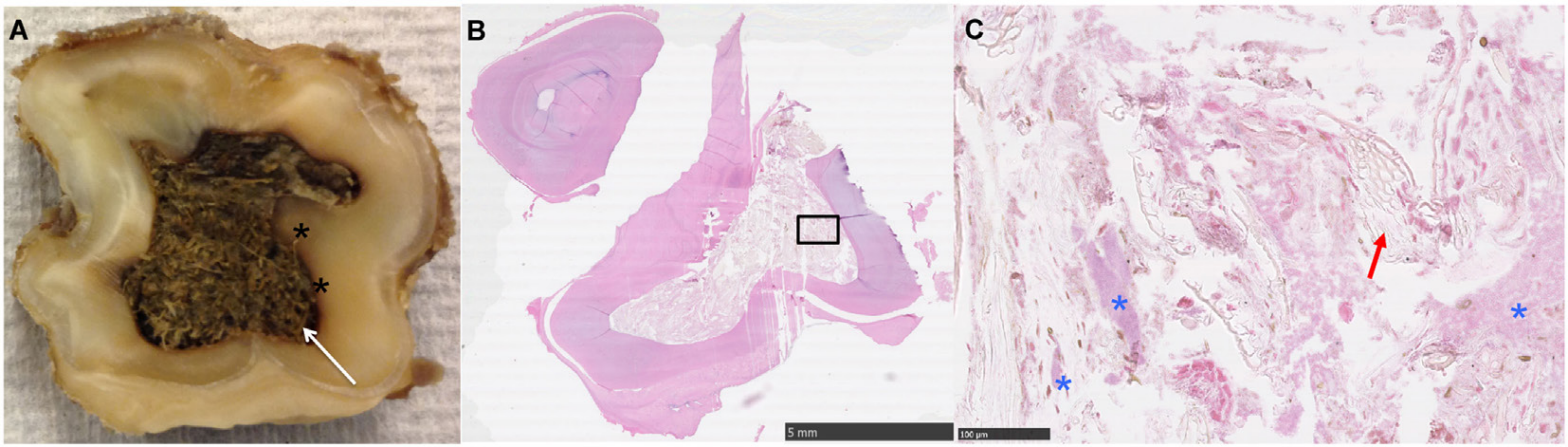
**(A)** Transverse section near the apical level of an apically infected Triadan 208: note the large amount of plant material within the common pulp cavity (white arrow) and staining of the adjacent dentine (*). **(B,C)** Histological image of transverse section of apex (HE): note the large pulp cavity containing plant material (red arrow) and bacteria (*).

Gross pulpar abnormalities including discolored, absent, or necrotic pulp were present in 23 of 28 teeth (81.5%), all which were later histologically confirmed to have pulpar/apical disease (see Histological Findings). Intrapulpar food material was present in 8/28 (28.6%) teeth (Figures 1A–C). The eight control teeth, one supernumerary, and the dysplastic tooth had no gross pulpar abnormalities.

#### Dentine

Gross dentinal changes, mainly the presence of circumpulpar dentinal staining and irregular dentinal–pulpar margins, were grossly identified around 1 or more pulp horns in 18/28 teeth (64.3%) (Figures 1, 4B and 7). A grossly appreciable communication was identified between a carious infundibulum and infected pulp horns in one maxillary tooth.

#### Periodontal Changes

Generalized periodontal thickening or fibrous soft tissue swellings of the periapical region were grossly identified in 25/28 teeth (89.3%) with apical infection. Limited, subgingival periodontal changes were visible in one dysplastic tooth without endodontic disease, likely due to malformation of the junction between the periodontal ligament and the alveolar bone.

#### Cementum

Proliferative calcified apical changes, including thickened, irregularly shaped roots and/or more generalized apical hypercementosis, were grossly identified in 24/28 teeth (85.7%) with apical infection. Limited peripheral cemental destructive changes were present near the gingival margin in the dysplastic cheek tooth.

#### Infundibulae

Gross and deep caries with discolored enamel (grade 2 caries) was identified in the infundibulae of 7/26 maxillary cheek teeth (26.9%), with as noted a pulpar–infundibular connection in one tooth.

In summary, of the 28 teeth later confirmed histologically to have pulpar/apical infection, 23 had gross pulpar abnormalities, 26 had apical calcified tissue changes, and 26 had periapical periodontal changes. Gross changes in external shape and of internal architecture (on cut section) were present in all three supernumerary (two with intercurrent apical infection and one endodontically normal) and the dysplastic tooth. Limited periodontal and peripheral cemental thickening were present in the dysplastic tooth. No gross changes were identified in the eight control teeth.

### Histological Findings

#### Alveolar Bone

**Figure 2:**
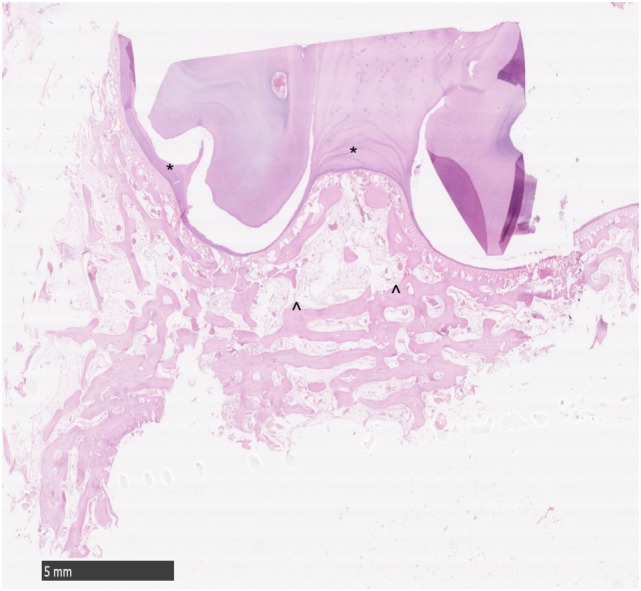
Histological transverse section of normal alveolar bone. HE. Note the smoothly outlined peripheral cemental profile (*) that is bordered more peripherally by anastomosing trabeculae of alveolar bone. The main bony trabeculae are oriented parallel to the profile of the circumference of the tooth (^^^) and are interspersed with marrow fat. No osteoclastic activity is apparent.

**Figure 3:**
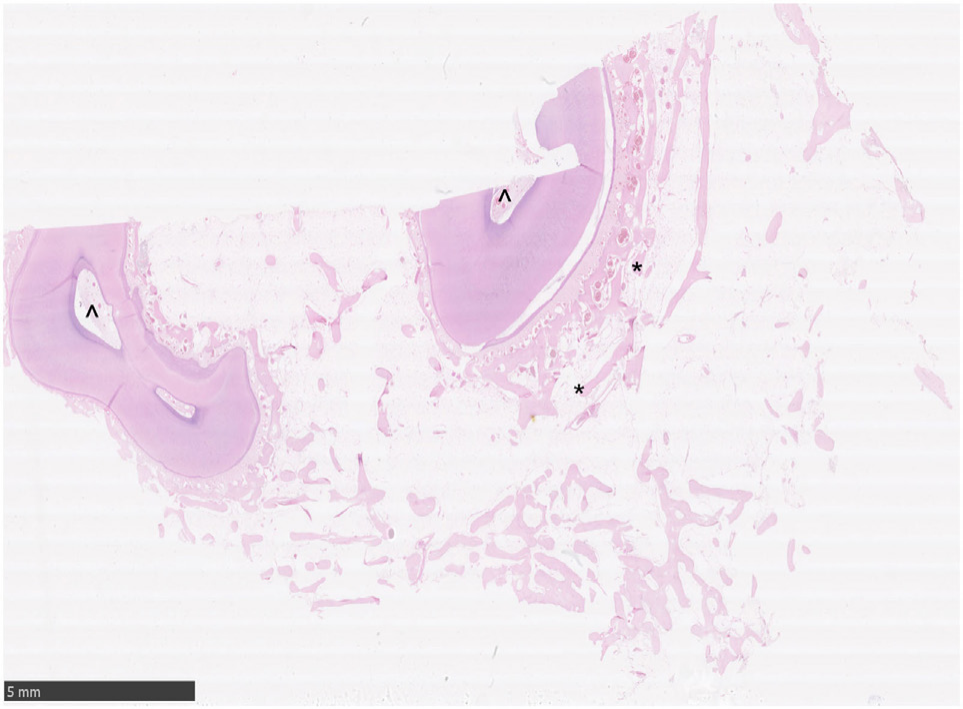
Histological transverse section of normal alveolar bone. HE. Note the alveolar bony trabeculae oriented parallel to the profile of the circumference of the tooth (*) and interspersed with marrow fat. No osteoclastic activity is apparent. Note the normal pulp stroma with blood vessels (^^^).

In the four control maxillary cheek teeth with attached alveolar bone, the peripheral cemental profile was smooth histologically and supported by a band of well-vascularized periodontal ligament of uniform thickness, in turn bordered more peripherally by anastomosing trabeculae of alveolar bone. The main bony trabeculae were oriented parallel to the circumference of the tooth and were interspersed with marrow fat (Figures 2 and 3). No osteoclastic activity was detected in the alveolar bone of any control teeth.

**Figure 4:**
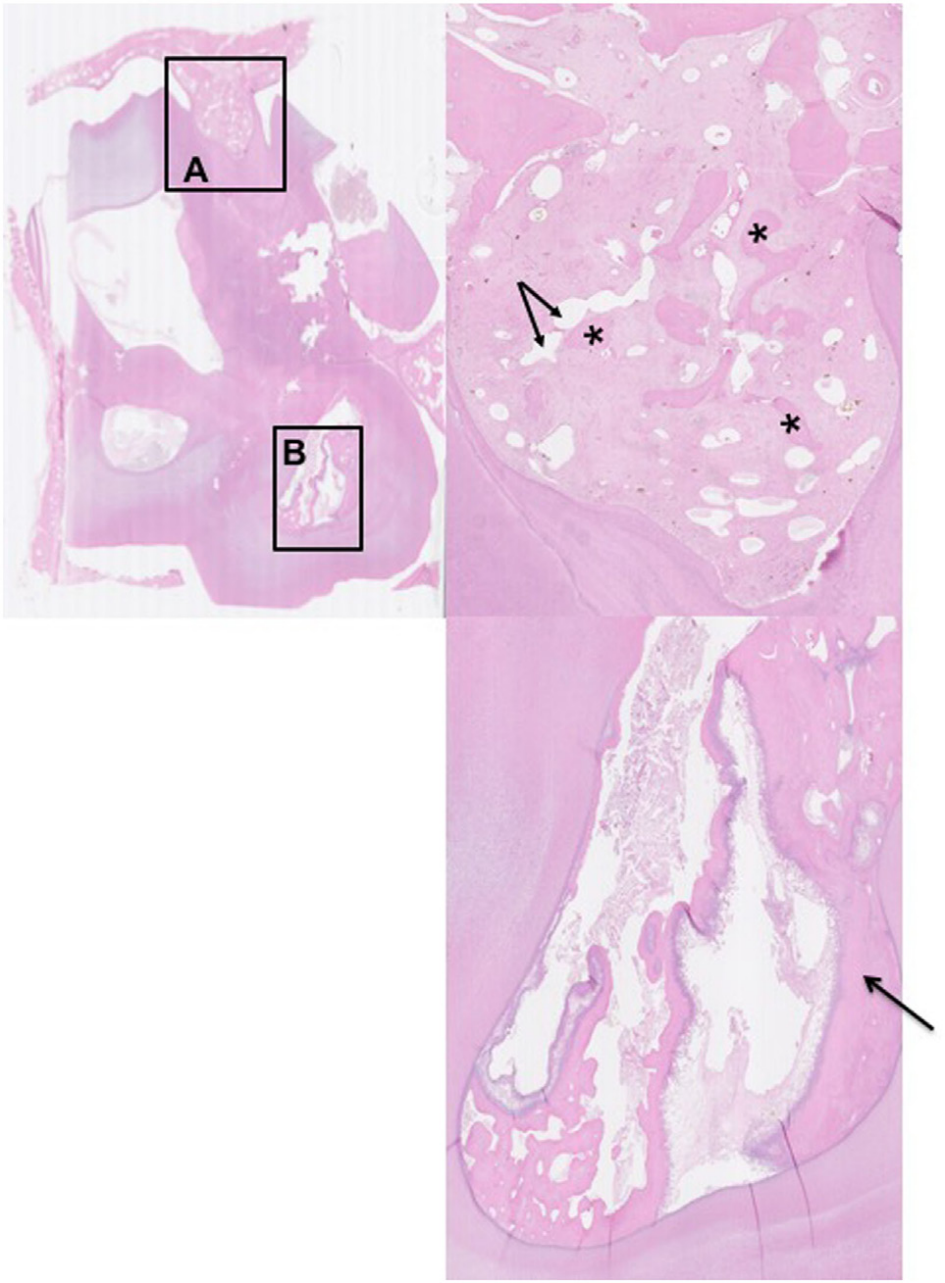
Histological transverse section of the apex of Triadan 110 including of distorted alveolar bone. HE. There is focal disruption and disorganization of the normal trabecular pattern of the alveolar bone with thickening of the surrounding periodontal ligament. The thickened periodontal ligament and disorganized islands of alveolar bone form a protruding “tongue” that focally displaces the peripheral cementum. **(A)** The thickened periodontal ligament is composed of increased amounts of mature collagen-containing enlarged, irregular islands of disorganized alveolar bone (*) and vascular structures (arrows). **(B)** Pulp containing debris and irregular secondary dentine: note the marked lysis within irregular secondary dentine (black arrow).

The 21 apically infected teeth with attached dental alveolus had changes in the alveolar bone that were not present in the control teeth. For the purposes of this study, these changes were regarded as abnormal. The changes were marked in 16 teeth and less severe in five. They were characterized by multifocal disruption of the normal trabecular pattern of the alveolar bone, which was usually associated with expansion/thickening of the periodontal ligament. Entrapped within this stroma were variably sized, irregular islands of disorganized alveolar bone, many of which had scalloped profiles associated with numerous osteoclasts occupying Howship’s lacunae (Figures 4A, 6A and 6B). There were also areas of increased cellularity composed of densely packed spindle cells consistent with fibroplasia (Figure 6B).

In the alveolar bone of the five teeth with milder changes, there was mild scalloping of the alveolar bone, mild osteoclast activity, and slightly increased thickening of the periodontal ligament. In three of these five cases, there was slight sclerosis of the periapical alveolar bone on both radiographic and CT imaging.

#### Periodontal Ligament

In addition to the aforementioned thickening of the periodontal ligament associated with alveolar bone changes in all 21 alveoli, further histological abnormalities were noted in the periodontal ligament that remained attached to 18/28 apically infected teeth (64.3%). These changes included the presence of hemorrhage/fibrin (*N* = 12/28) (43%), infiltration with bacteria/neutrophils (*N* = 9/28), plasma cells (*N* = 3/28), and lymphocytes (*N* = 1/28).

#### Pulp

Pulpar changes were histologically present in all 28 teeth diagnosed with pulpar/apical infection, and such histological evidence was the main criterion for diagnosing these teeth with pulpar/apical infection. These changes involved 96 of the 152 pulps (mean 3.6 affected pulps/tooth, range 1–5) in the 28 teeth, with every pulp diseased in 3/28 teeth.

Histological pulpar abnormalities included what we termed “faded pulp” in 20/28 (71.4%) teeth. This feature was characterized by a pulp stroma that was much paler than that of normal viable pulps, with loss of nuclear detail in stromal fibroblasts, loss of normal vasculature and, sometimes, the accumulation of loose dentinal debris at the pulp periphery (9). Also recognized were the presence of intrapulpar bacteria and neutrophils (*N* = 19/28), food material (*N* = 8), and pulp necrosis (*N* = 1). Pulp stones (*N* = 1–5) were present within the pulp and secondary dentine in 8/28 apically infected and in 4/8 control teeth. All pulps in the eight control teeth, one supernumerary tooth and the dysplastic tooth were histologically normal.

#### Dentine

Histological changes were present in dentine surrounding infected pulps in 7/28 (25%) infected teeth, including dentinal lysis at the pulp periphery (*N* = 5) and irregular pulpar margins (*N* = 2). Five of the seven teeth with histological pulpar changes showed gross dentinal abnormalities. Tertiary dentine was not identified in any teeth.

#### Peripheral Cementum

Histological abnormalities were present in the peripheral periapical cementum in 15/28 apically infected teeth, including cemental lysis and caries-like destruction (*N* = 5), plaque/biofilm formation (*N* = 13), and intra-cemental abscess formation and bacteria (*N* = 5).

#### Infundibulae

Histological infundibular changes were found in 9/26 (34.6%) maxillary cheek teeth including the accumulation of plant material, bacteria, debris, and carious infundibular cementum.

The final pathological diagnoses were apical infection (*N* = 28) including in two supernumerary teeth; a further (uninfected) supernumerary tooth and a dysplastic tooth.

#### CT Findings (Pre-Extraction at 1.5 and 3 mm Intervals)

No pulpar or periapical abnormalities were detected in one supernumerary tooth, the dysplastic tooth, or the eight control teeth. In addition, one tooth with pulpar discoloration and histological evidence of neutrophil/bacterial pulpar infiltration did not have any CT imaging evidence of apical infection.

The main CT findings in the 28 teeth diagnosed with apical infection included: gas within pulps (*N* = 19/28) (67.9%); irregular pulp horn margins (*N* = 16); increased pulpar volume (*N* = 7) [total of 19/28 teeth (67.9%) with pulpar changes]; widened periodontal space (*N* = 5); gas within periapical tissues (*N* = 2); root clubbing (*N* = 20), root fragmentation (*N* = 7); periapical halo (*N* = 4); periapical bone changes including peripheral sclerosis (*N* = 20); alveolar bone thickening (*n* = 3); resorption/lysis more axially (*N* = 8), and infundibular changes (other than central cemental hypoplasia) (*N* = 12). The three supernumerary and the dysplastic tooth all had abnormal internal architecture of their calcified dental tissues.

In all 16 teeth with severe histological alveolar bone changes, perialveolar bone changes were visible on CT, but only 4 of these 16 cases had identifiable periapical alveolar changes radiographically.

#### CT Findings (Post-Extraction 0.5 mm Intervals)

Following extraction, CT imaging of the 28 teeth diagnosed with apical infection at 0.5 mm slice thickness also showed convincing evidence of apical infection on CT imaging in 27/28 teeth, including the presence of intrapulpar gas in 17/27 teeth (2 teeth with gas in pulps on pre-extraction CTs sustained iatrogenic fractures to the roots of the affected pulp horns during extraction with exposure of the affected pulp and loss of intrapulpar gas); increased pulpar volume in 7/27 teeth and irregular pulp horn margins in 14/27 teeth [total of 21/28 (75%) with pulpar changes]. Pre- and post-extraction CT imaging changes in infundibulae and roots were similar.

### Radiography

Radiographic abnormalities strongly indicative of apical infection were found in 14 of the 28 apically infected teeth (50%), including root blunting (clubbing) (*N* = 14/27) and alveolar bone sclerosis (*N* = 8/27) (Figure 8), with no convincing radiographic changes present in 13 teeth (Figure 9).

## Discussion

Liuti et al. (9) have recently shown CT to be highly sensitive (97%) in detecting maxillary cheek teeth periapical infection, as assessed by gross pathological and histological evaluation of teeth diagnosed with apical infections by CT and subsequently extracted on clinical grounds. A similar sensitivity (96.4%) was found in this study during which CT imaging of cadaver-sourced maxillary and mandibular teeth identified 27 of 28 teeth with pathological evidence of apical infection. This study recorded a single false negative case (1/28) on CT imaging, in contrast to Liuti et al. (9) where a single false positive case (1/32) was recorded on CT imaging.

A significant amount of dental imaging information is also gained from assessment of the periodontal ligament and alveolus of a suspect tooth. In clinical cases, the periodontal ligaments can only be pathologically examined in areas that (very variably) remain attached to the extracted tooth. In addition, some periodontal pathological changes, such as hemorrhage and fibrin, may be iatrogenic due to the *in vivo* extraction process (9). It is interesting that in the current *ex vivo* study where iatrogenic extraction-related periodontal fibrin deposition or intra-periodontal hemorrhage could not occur, hemorrhage and/or fibrin were still present in the periodontal membranes of 43% (12/28) apically infected teeth. This is in contrast to the 59% (19/32) periodontal hemorrhage/fibrin deposition from teeth extracted *in vivo* (9), where the higher prevalence of intra-periodontal changes was likely due to iatrogenic extraction damage.

Alveolar bone cannot be pathologically examined in clinical cases, so it is not possible to compare imaging and pathological alveolar bone findings in such cases. The use of diseased teeth from fresh cadaver heads in this study allowed most of the periodontal ligament to be removed along with adjacent alveolar bone in 70% (21/30) of teeth. This facilitated pathological assessment of alveolar bone when present and, as noted, allowed a more complete assessment of the periodontal ligaments that additionally, were free of iatrogenic extraction-related inflammatory changes.

Alveolar bone was histologically abnormal around all 21 apically infected teeth where it remained attached. Changes included extensive osteoclastic activity and marked disruption of the trabecular alveolar bone with its replacement by a collagenous stroma, the latter leading to apparent thickening of the periodontal membranes on CT imaging.

Normal alveolar bone constantly remodels to accommodate the changing size and shape of erupting brachydont teeth (18), and this phenomenon is more pronounced in hypsodont teeth that have more prolonged eruption. However, the above-noted changes in the periapical alveolar bone of the infected teeth greatly differed from those in the alveolar bone of the control teeth alveoli.

**Figure 5:**
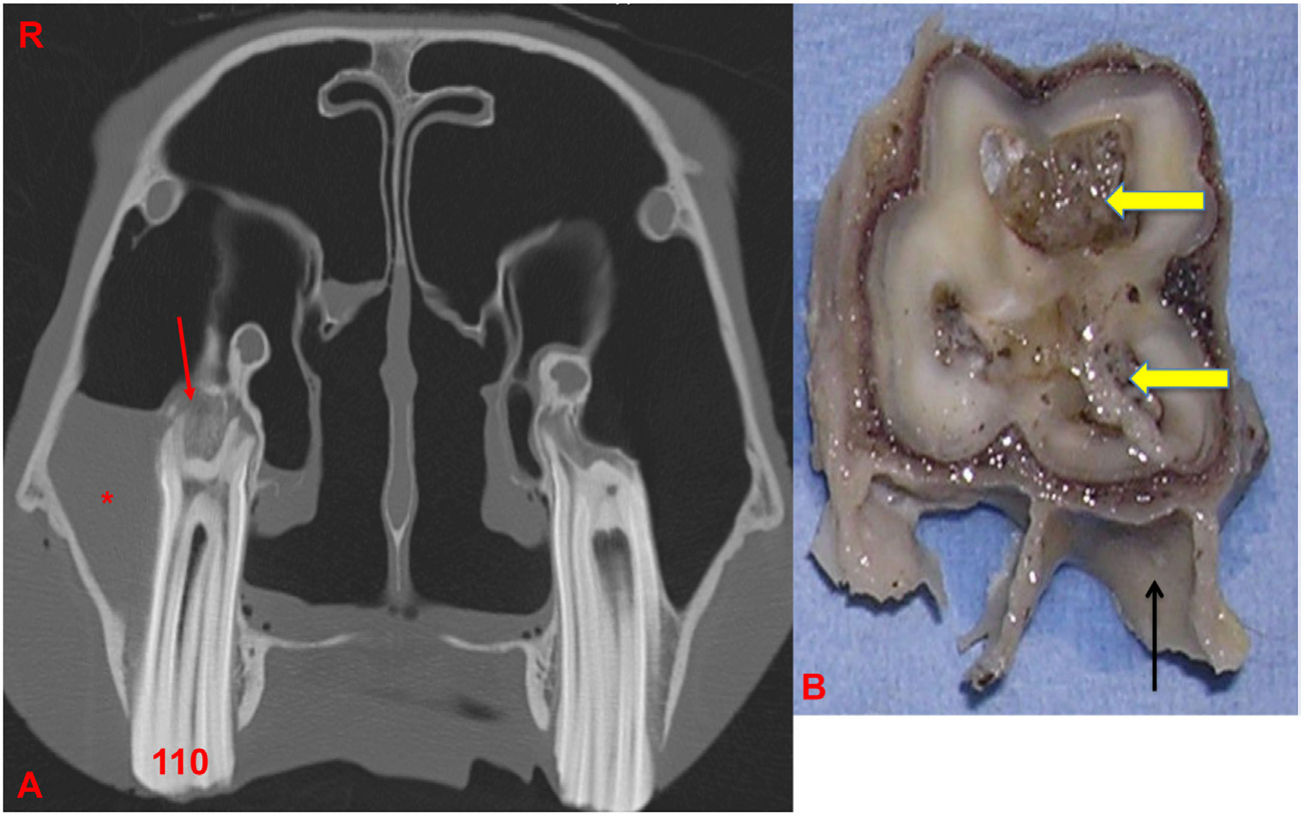
**(A)** Transverse CT image at the level of 110: note the thickening, sclerosis, and also, erosion of the alveolar bone (red arrow) with apical clubbing. A moderate amount of fluid (*) is present within the caudal maxillary sinus. **(B)** Transverse section of 110 at the level of the apex: note the large and discolored pulps (arrows) and adjacent dentine: alveolar bone is present in this section (black arrow). R, right.

Buhler et al. (14) reported alveolar bone sclerosis as a CT finding with equine periapical infection. In this study, sclerosis was detected with CT imaging, especially on the abaxial aspect of the periapical alveolar bone in 20/28 (71.4%) cases of apical infection, with thickening of this bone less commonly recognized (3/28). By contrast, alveolar bone resorption or lysis was recognized on CT imaging, especially on the axial aspect of alveolar bone in 28.6% (8/28) of cases, sometimes immediately adjacent to areas of sclerosis (Figures 5 and 7).

**Figure 6:**
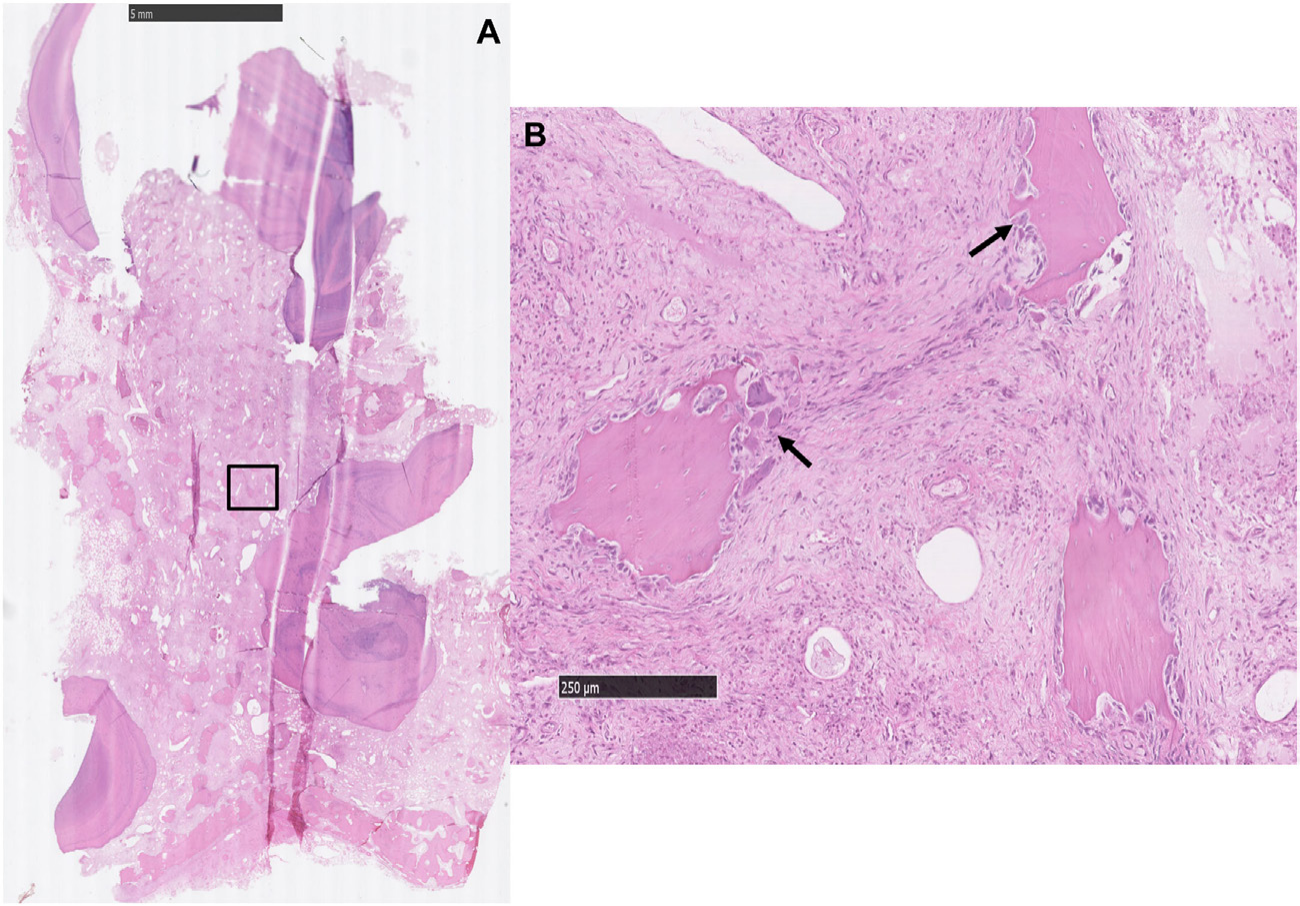
**(A)** Histological transverse section of the periapical aspect of an apically infected tooth where the soft tissue between the roots is expanded by a large amount of collagen-containing, entrapped, variably sized, irregular islands of alveolar bone [inset **(B)**]. HE. **(B)** The stroma contains entrapped islands of alveolar bone with scalloped borders and numerous osteoclasts (black arrows) occupying bays (Howship’s lacunae). There is also moderate to marked fibroplasia in the intervening stroma. HE.

**Figure 7:**
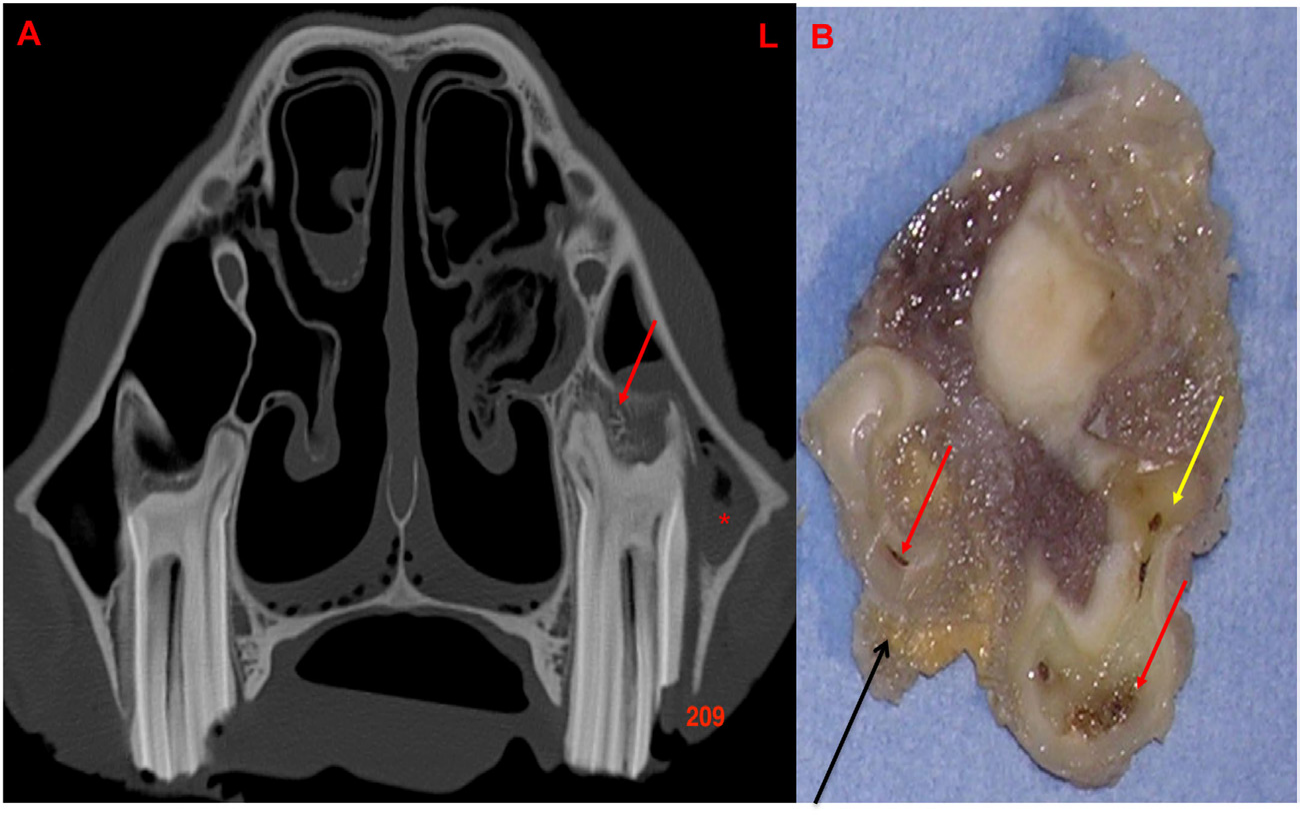
**(A)** Transverse CT image at the level of 209 with a lateral idiopathic “slab” fracture (through first and second pulp horns): note the thickening, sclerosis, and also erosion of the alveolar bone (red arrow) with apical clubbing. A moderate amount of fluid (*) is present within the rostral maxillary sinus. **(B)** Transverse section of 209 at the level of the apex: note the large and discolored pulps (red arrow) and discolored dentine (yellow arrow): alveolar bone is present in this section (black arrow). L, left.

**Figure 8:**
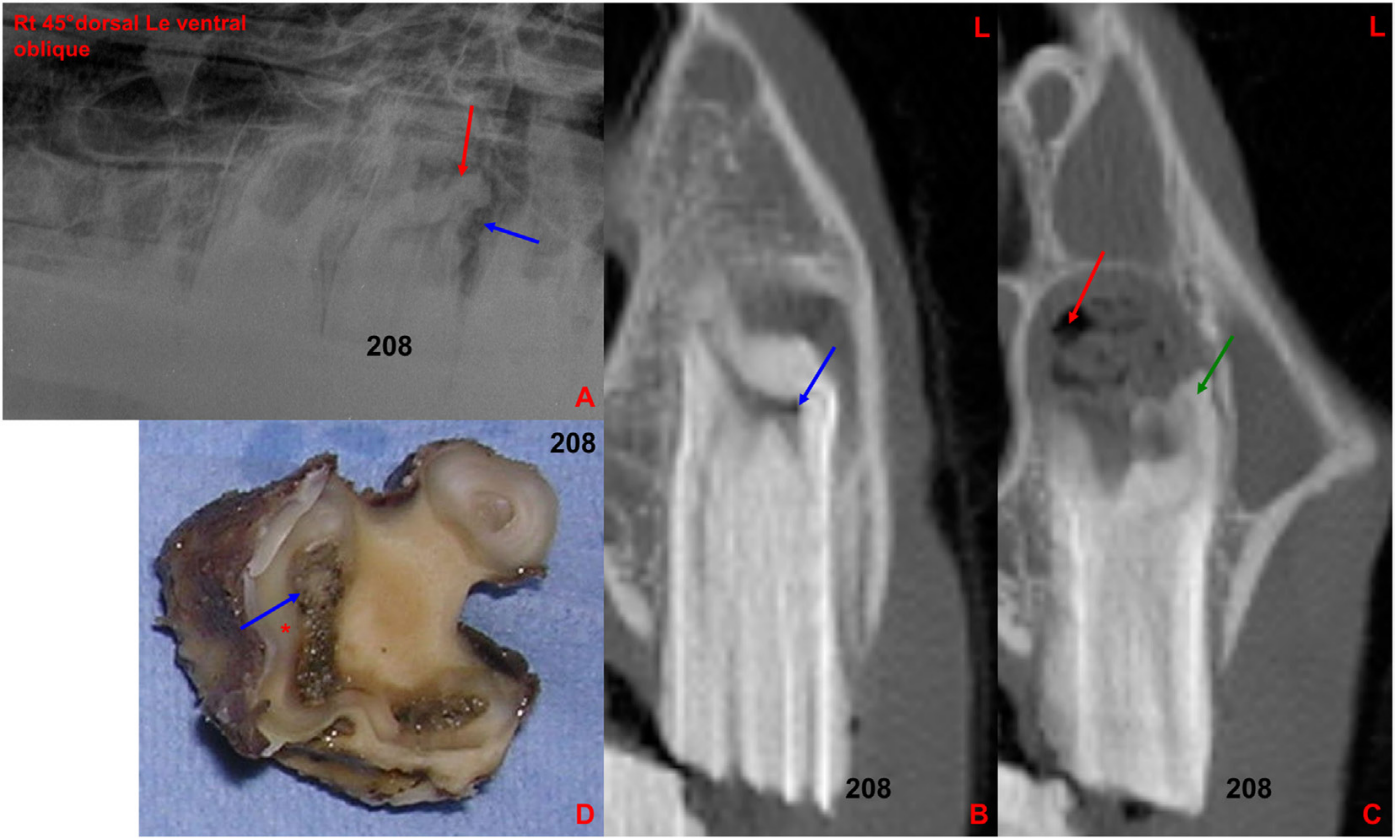
**(A)** Right 30°dorsal left ventral oblique radiographic projection of the rostral maxillary cheek teeth: note the marked clubbing of the caudo-buccal 208 tooth root (red arrow) with an irregular surrounding lucency (blue arrow), both indicative of apical infection. **(B)** Transverse CT image at the level of 208: note small gas-attenuating structures (blue arrow) within the buccal aspect of the common pulp chamber. **(C)** Transverse CT image at the level of 208 [more caudally located than image **(B)**]: note the increased size of the periapical space, clubbing of a buccal root (green arrow) and multiple gas pockets (red arrow) mixed with soft tissue attenuating structures in the perialveolar space. **(D)** Transverse section of the above extracted 208 at the apical level: note the large and discolored necrotic common pulp (blue arrow) and adjacent dentine (*). L, left.

**Figure 9:**
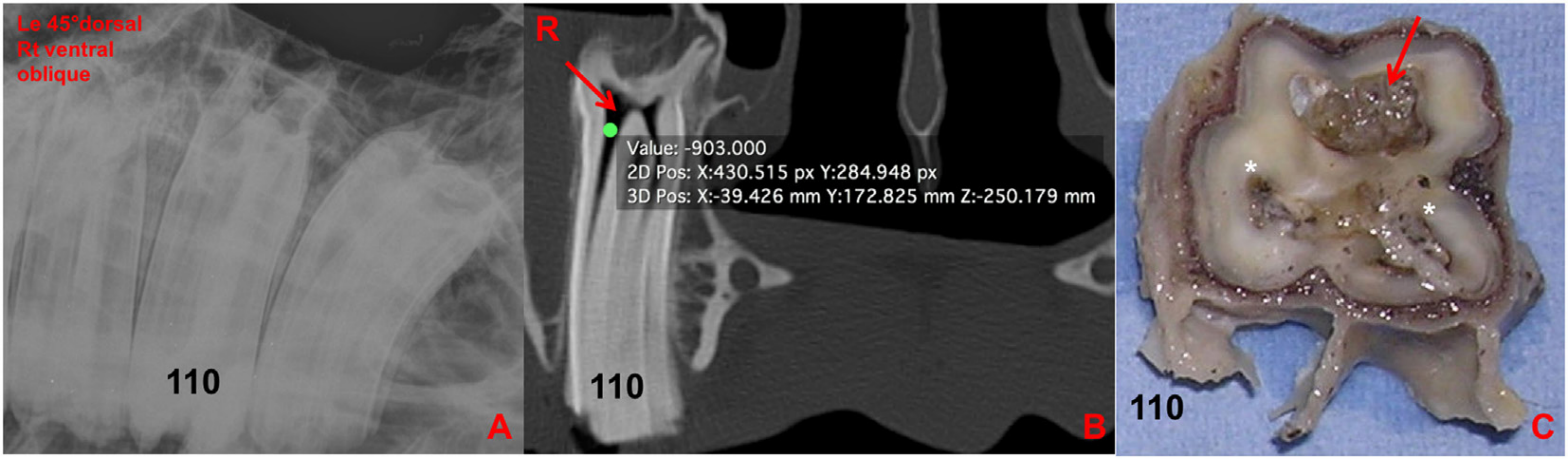
**(A)** Left 30°dorsal right ventral oblique radiographic projection at the level of 110. No clinically significant abnormalities were detected in this radiograph. **(B)** Transverse CT image at the level of 110: note the large amount of gas-attenuating structures (red arrow) within the common pulp chamber and pulp horns (−903 HU). **(C)** Transverse section of 110 at the level of the apex: note the discolored large common pulp chamber of the buccal pulp horns (red arrow) and the other two discolored pulp horns and adjacent dentine (*). R, right.

Radiographically, alveolar bone sclerosis has been described as an imaging feature dental apical infection (6) and was detected in 8/28 apically infected cases in this study, 4 of which contained alveolar bone changes histologically.

Apically infected teeth always have one or more infected pulps (that precedes the periodontal infection) (19). All 28 apically infected teeth in this study had pulpar abnormalities confirmed microscopically, while 68% had grossly evident pulpar abnormalities. Subsequent progressive bacterial destruction of the surrounding dentine creates larger pulp horns that also may develop irregular pulpo-dentinal margins (9, 14). Such pulp horn widening or irregular margins were detected by CT in 19 out of 28 (67.9%) apically infected cheek teeth in this study.

Other CT changes associated with apical infection, including clubbing (hypercementosis), fragmentation, and/or lysis of roots, were present in 27/28 (96.4%) of the cases. Changes to the apical calcified tissues (mainly increased deposition of cementum around roots) were more obvious grossly and were present in 26/28 (92.8%) teeth while histological changes (including erosion, necrosis, and biofilm/plaque deposition) were present in 15/28 (53.6%). This discrepancy between gross and histological findings may be due to loss of some roots during decalcification and histological processing (9). It has been reported (20) that peripheral cemental changes in apically infected mandibular cheek teeth were subtle and included cemental discoloration or loss, or increased cemental deposition.

Casey et al. showed that CT and pathological findings in formalin-preserved, apically infected cheek teeth correspond well with respect to mineralized dental tissue changes or the presence of food material in the pulp canals (20). However, pulpar changes that were evident grossly and histologically were not detectable on CT (20). By contrast, *in vivo* CT studies on clinical cases of apical infection (9, 13, 14, 16) have found CT to be very effective for identifying dental soft tissue (pulpar and periodontal) changes in diseased teeth, and particularly for recognizing gas in pulpar and periapical periodontal infections.

The production of gas by anaerobic bacteria, such as *Clostridium perfringens* (the cause of gas gangrene) where infected wounds become grossly emphysematous, is well recognized. Likewise, the presence of gas-attenuating areas in the pulp and/or periapical soft tissues of apically infected cheek teeth could be attributed to gas production by anaerobic bacteria (whose metabolism involves gas production). Such infection could occur following blood or lymphatic borne (anachoretic) or direct routes of apical infection from the oral cavity, including dental fractures, infundibular caries, or deep periodontal disease (periodontal–endodontic lesion) (21).

With severe (grade 3) infundibular caries with subsequent erosion of cementum, enamel and dentine, and pulpar invasion, or with infundibular caries-related sagittal fractures and subsequent apical infection (21–24), the presence of gas-attenuating areas within the infected pulp or periapical areas could additionally be attributed to the movement of gas (including air) from within the affected infundibulae to the pulp. With idiopathic fractures of the clinical crown and secondary apical infection (25, 26), air from oral cavity could also contribute to gas attenuation of infected pulpar/periapical tissues.

Casey et al. (20) compared post-extraction CT images of formalin-preserved mandibular cheek teeth with their gross pathological and histological but did not detect gas attenuation of infected pulps.

It is possible that the prolonged storage of teeth by Casey et al. (20) in formalin solution before CT may have affected the stability or preservation of intrapulpar gas, although apically infected teeth stored in formalin in this institute for over 4 years have still contained intrapulpar gas (authors’ personal observations). In this study, intrapulpar gas was recognized on CT in 19/28 (67.9%) of infected teeth, while an *in vivo* study showed intrapulpar gas in 100% of apically infected teeth (9). The lower prevalence of intrapulpar gas in this study may be related to freezing of the heads before imaging examinations that could have affected the later distribution and/or recognition of intrapulpar gas on CT imaging.

Using the HU values from CT images is an established methodology to identify small gas cavities in tissue; vacuum phenomenon in the intervertebral disks has been described on CT as an accumulation of gas (principally nitrogen) in the vertebral column with a range of density between −900 and −980 HU (27).

None of the infected teeth had advanced gross destructive changes or extensive proliferative cemental apical deposits, i.e., all appeared to be low-grade chronic pulpar/apical infections and this may explain the poor sensitivity of radiography in diagnosing these infections at this early stage. In a similar study (9), the current authors found that only 13% of apically infected teeth had grossly appreciable abscesses of their apices, while 67% of apically infected teeth had some remaining viable pulps, supporting the presence of a chronic low-grade infection of the periapical region in that study. In this study, a higher proportion of teeth, i.e., (89%) 25/28, had some remaining viable pulps.

Radiography detected changes including tooth root clubbing (52%) and periapical sclerosis (29.6%) in teeth with histologically confirmed apical infection. Overall, radiography detected changes that were considered sufficient to recommend extraction in 14/28 (50%) of the current cases, similar to the findings of Liuti et al. (9) where 17/30 (53%) of teeth contained what could be termed definitive evidence of apical infection, albeit at an early stage of infection. With progression of these apical infections, radiography would likely become more sensitive in detecting the pulpar/apical infection.

Infundibular caries can cause midline sagittal fractures of affected teeth (25, 26), and extension of infundibular caries can also cause maxillary cheek teeth pulpar/apical infection (21, 24) as was found in one tooth in this study. In this study, histological evidence of infundibular caries was present in 37.5% of all maxillary cheek teeth, with a poor correlation between pathological and CT findings. Anatomical (28) and imaging (15) studies of cheek teeth infundibulae in clinically normal horses showed that 88–90% of infundibulae are incompletely filled with morphologically normal cementum, with these defects preferentially affecting the Triadan 09 position in older horses. Suske et al. (24) found infundibular cemental hypoplasia in 51% of normal cheek teeth and in 72% of cheek teeth affected by periapical and endodontic infections, fractures, or periodontal disease. In fact, infundibular cemental hypoplasia and secondary infundibular cemental caries are so common that interpretation of infundibular images is problematic (9). Unless there is a midline sagittal infundibular caries-related fracture or evidence of extension of infundibular caries to enamel or dentine, such lesions are generally considered to be clinically insignificant at that time, although they may later progress to cause fracture and/or pulpitis/apical infection.

Apically infected teeth have by definition, changes in their periodontal ligaments, especially around the apex, and periodontal lesions were grossly identified in 89% (25/28) of apically infected teeth in this study. However, periodontal lesions were histologically detectable in only 64.2% (18/28) of these 28 teeth. The absence of this finding in the other 10 teeth is likely due to failure to capture localized periapical periodontal lesions in sections, loss of apical tissues during histological preparation or possibly due to the pulpitis not extending to the periapical periodontal tissues in some very early pulpar infections. There was a good correlation in this study between the histological findings and radiographic, CT and gross periodontal findings. However, it can be difficult to differentiate grossly between preexisting changes and extraction-induced changes in the periodontal ligament. In this study, this was not of concern because the alveolar bone and periodontal ligament were removed with the tooth after death.

Inflammatory cells were only present in a small number of periodontal tissues from these teeth and they mainly had a perivascular distribution, suggesting hematogenous origin rather than direct extension from an apical lesion. Gross examination excluded a descending periodontal route of infection for the two infected supernumerary teeth. Such a route can come into play in some supernumerary/dysplastic teeth that are not well seated into their alveoli (29), as was the case for the dysplastic tooth in this study.

In this study, pulp stones were found both free in the pulp and embedded in the secondary dentine, both in abnormal (16/30) and control teeth (4/8) similar to the findings of Liuti et al. (9). For this reason, their presence was not considered as an abnormality.

This study has shown an inexplicably high prevalence (51.9%) of pulpar/apical infection in 54 heads obtained from a rendering plant. Unfortunately, no clinical histories were available for any of these cases, and this extraordinarily high prevalence of dental disease may be a reflection of the study population. These were all ill horses that had died from disease or were euthanized on humane grounds. It would appear that none was euthanized on the grounds of old age since the mean age was 12 years (5–20 years). No heads contained evidence of significant decomposition and, in all 26 horses with the 28 apically infected teeth, just 4.5% (28/627) of the cheek teeth had imaging or gross pathological evidence of apical infection. In addition, in 89% (25/28) of the apically infected teeth, some pulp horns were grossly and histologically normal, thus confirming that postmortem changes did not cause the observed pathological changes.

The true prevalence of clinical equine cheek teeth endodontic/periapical disease in the general equine population is unknown but, as adjudged by the caseload of this equine hospital, cases with external signs of apical infection such as mandibular or maxillary swellings, sinus tracts or dental sinusitis, it is likely to be less than 2%. However, occlusal pulpar exposure (indicating some prior pulpar insult) is sometimes clinically apparent in teeth without clinical signs of apical infection. In addition, some cheek teeth with “idiopathic fractures” (1) have a reduced secondary dentinal thickness, which is also evidence of prior pulpar disease (25). Consequently, pulpitis and non-clinically detected periapical infection may be more common than currently realized, especially in systemically ill horses, although it may only affect some of the pulp horns of a single cheek tooth in most horses.

## Conclusion

Changes highly indicative of apical infection were identified on CT imaging in 96.4% and radiographically in 50% of pathologically confirmed apically infected cheek teeth. Alveolar bone changes were histologically present in all 21 examined alveoli, and all 16 alveoli with marked alveolar bone changes had detectable CT alveolar changes. This study confirms the superior accuracy of CT compared with radiography in detecting equine cheek tooth apical infection and showed excellent correlation between both dental and alveolar CT imaging and pathological findings.

## Ethics Statement

This study was approved by the Ethical Review Committee of The Royal (Dick) School of Veterinary Studies, Roslin Institute, The University of Edinburgh on the 16th February 2012.

## Author Contributions

TL contributed to study design and execution, data analysis and interpretation, and manuscript preparation. SS contributed to study execution and interpretation and manuscript preparation. PD contributed to study design and execution, data analysis, and manuscript preparation. All the authors approved the final manuscript.

## Conflict of Interest Statement

The authors declare that the research was conducted in the absence of any commercial or financial relationship that could be constructed as a potential conflict of interest.
